# The Impact of lncRNAs in Diabetes Mellitus: A Systematic Review and *In Silico* Analyses

**DOI:** 10.3389/fendo.2021.602597

**Published:** 2021-03-19

**Authors:** Cristine Dieter, Natália Emerim Lemos, Nathalia Rodrigues de Faria Corrêa, Taís Silveira Assmann, Daisy Crispim

**Affiliations:** ^1^Endocrine Division, Hospital de Clínicas de Porto Alegre, Porto Alegre, Brazil; ^2^Post-Graduate Program in Medical Sciences: Endocrinology, Faculdade de Medicina, Universidade Federal do Rio Grande do Sul, Porto Alegre, Brazil

**Keywords:** lncRNAs (long non-coding RNAs), type 1 diabetes mellitus (DM1), type 2 diabetes mellitus (T2DM), systematic review, target prediction

## Abstract

Long non-coding RNAs (lncRNAs) are non-coding transcripts that have emerged as one of the largest and diverse RNA families that regulate gene expression. Accumulating evidence has suggested a number of lncRNAs are involved in diabetes mellitus (DM) pathogenesis. However, results about lncRNA expressions in DM patients are still inconclusive. Thus, we performed a systematic review of the literature on the subject followed by bioinformatics analyses to better understand which lncRNAs are dysregulated in DM and in which pathways they act. Pubmed, Embase, and Gene Expression Omnibus (GEO) repositories were searched to identify studies that investigated lncRNA expression in cases with DM and non-diabetic controls. LncRNAs consistently dysregulated in DM patients were submitted to bioinformatics analysis to retrieve their target genes and identify potentially affected signaling pathways under their regulation. Fifty-three eligible articles were included in this review after the application of the inclusion and exclusion criteria. Six hundred and thirty-eight lncRNAs were differentially expressed between cases and controls in at least one study. Among them, six lncRNAs were consistently dysregulated in patients with DM (*Anril*, *Hotair*, *Malat1*, *Miat*, *Kcnq1ot1*, and *Meg3*) compared to controls. Moreover, these six lncRNAs participate in several metabolism-related pathways, evidencing their importance in DM. This systematic review suggests six lncRNAs are dysregulated in DM, constituting potential biomarkers of this disease.

## Introduction

Diabetes mellitus (DM) is a group of metabolic disorders that have in common the chronic hyperglycemia, which results from defects in insulin secretion, insulin action, or both (1). Accordingly to the International Diabetes Federation Atlas 2019, an estimated 463 million adults are currently living with DM (9.3% of the world population), and this number is projected to reach 700 million by 2045 (2). Thus, DM has achieved epidemic proportions worldwide, being associated with increased morbidity and mortality rates due to its specific micro- and macrovascular complications (1, 2).

Type 1 DM (T1DM) accounts for 5–10% of all DM cases and usually appears in people younger than 30 years (1, 2). T1DM is an autoimmune disease caused by the progressive destruction of pancreatic beta-cells by macrophages and T lymphocytes, making patients insulin-dependent for life (1, 3). Type 2 DM (T2DM) comprises 90–95% of worldwide diabetic cases and generally arises in subjects older than 40 years and with obesity. Hyperglycemia in T2DM patients is caused by insulin resistance associated with different degrees of a relative beta-cell failure (1, 2). It is well known that susceptibility for both T1DM and T2DM is triggered by a multifaceted interaction among several environmental, genetic, and epigenetic factors (4–8).

Epigenetic factors regulate the complex crosstalk between genes and environmental factors without altering the DNA sequence and include DNA methylation, histone posttranslational modifications, and non-coding RNAs (ncRNAs) (7, 8). NcRNAs are regulatory RNAs that typically lack protein-coding capacity and play key roles in both physiological and pathological processes (9, 10). According to their length and functions, ncRNAs can be classified into different subtypes, including the long ncRNAs (lncRNAs), which are those ncRNAs with >200 nucleotides in length (10, 11).

LncRNAs can be located in the nucleus or cytoplasm and exhibit more specific expression profiles than mRNAs, being expressed in cell/tissue-, developmental stage-, or disease state-specific manners (10, 12, 13). A number of studies have suggested lncRNAs participate in several molecular processes involved in gene regulation, including epigenetic, transcriptional, and post-transcriptional regulation, through interaction with chromatin-remodeling complexes, binding to transcription factors or regulation of mRNA-binding proteins and microRNAs (another class of ncRNAs) (10, 14–16).

In this context, growing evidence has shown lncRNAs play key roles in regulating beta-cell function, apoptosis, insulin secretion, glucose metabolism, and insulin resistance (10, 17–22). Accordingly, a number of studies have reported changes in lncRNA expressions in patients with DM or in murine models of T1DM or T2DM (10, 23–29). Thus, lncRNAs are likely to be novel potential biomarkers for early diagnosis and prognosis of T1DM or T2DM (10, 29). For example, Carter et al. showed GAS5 might be a prognostic biomarker for T2DM since this lncRNA was decreased in serum of patients with DM from a US military veterans cohort (23). Individuals with lower *GAS5* expression were almost 12× more likely to have T2DM (23). Li et al. reported *ENST00000550337.1* upregulation in blood had high diagnostic value for identifying pre-DM and T2DM in patients from a Chinese cohort (25).

Therefore, to further investigate which lncRNAs may be involved in DM pathogenesis and used as potential biomarkers of this disease, we performed a systematic review of the literature on the subject. Moreover, bioinformatics analyses were performed to investigate the regulatory and functional roles of dysregulated lncRNAs in DM pathogenesis.

## Materials and Methods

### Search Strategy, Eligibility of Studies, and Data Extraction

This systematic review was designed and described in accordance with current guidelines (30, 31), and its protocol was registered at PROSPERO (http://www.crd.york.ac.uk/PROSPERO), under the identification: CRD42019124368. PubMed and EMBASE repositories were searched to retrieve all articles that investigated lncRNA expressions in T1DM or T2DM patients compared to non-diabetic controls. The research question was constructed based on the PICOS strategy (31), as follows: P (Population): patients with T1DM or T2DM; I (Intervention): lncRNA expression; C (Comparators): healthy control groups; O (Outcomes): DM; S (Study designs): case–control study, cross-sectional or cohort. The following medical subject headings (MeSH) were used: (“diabetes mellitus” OR “diabetes mellitus, type 1” OR “diabetes mellitus, type 2”) AND (“RNA, long noncoding” OR “untranslated RNA”). The search was restricted to English, Spanish, or Portuguese language papers and was finished on April 2020. Reference lists from all included articles were also manually reviewed in order to identify other relevant citations. Moreover, studies were also searched in the GEO database (https://www.ncbi.nlm.nih.gov/geo/).

We included original articles that analyzed lncRNA expressions in patients with T1DM or T2DM (cases) and subjects without DM (controls). Studies that did not have an appropriate control group were excluded. Two researchers (CD and NL) independently reviewed titles and abstracts of all articles to evaluate if they were eligible for inclusion in this systematic review.

Results were independently collected by two investigators (CD and NL) using a standardized abstraction form (31). Discrepancies between investigators were solved by discussion between them and, when necessary, a third reviewer (DC) was consulted. The following information was collected from each study included in this review: 1) characteristics of studies and samples; 2) information regarding lncRNA expressions, quantification method, analyzed tissue, and number of lncRNAs investigated; and 3) lncRNA expression profile in case and control groups.

### Evaluation of lncRNA Putative Target Genes and Functional Enrichment Analysis

Potential target genes for the consistently dysregulated lncRNAs in DM were searched using lncRNA2Target v2.0 (32) and starBase (33). The criteria for selecting the consistently dysregulated lncRNAs were: 1) lncRNAs with concordant results in ≥75% of the studies in which they were analyzed; and 2) lncRNAs analyzed in at least three studies. Statistical significances were reported after Benjamini–Hochberg (q-*value*) corrections for multiple comparisons (34). To better understand the biological relevance of lncRNA target genes, a network analysis was executed using PathDIP (accessed 23^th^ April 2020) (35). The nomenclature of mRNAs and lncRNAs were unified based on HUGO gene nomenclature committee (HGNC) and LNCipedia v5.2, respectively.

## Results

### Literature Search and Characteristics of Eligible Studies

**Figure 1:**
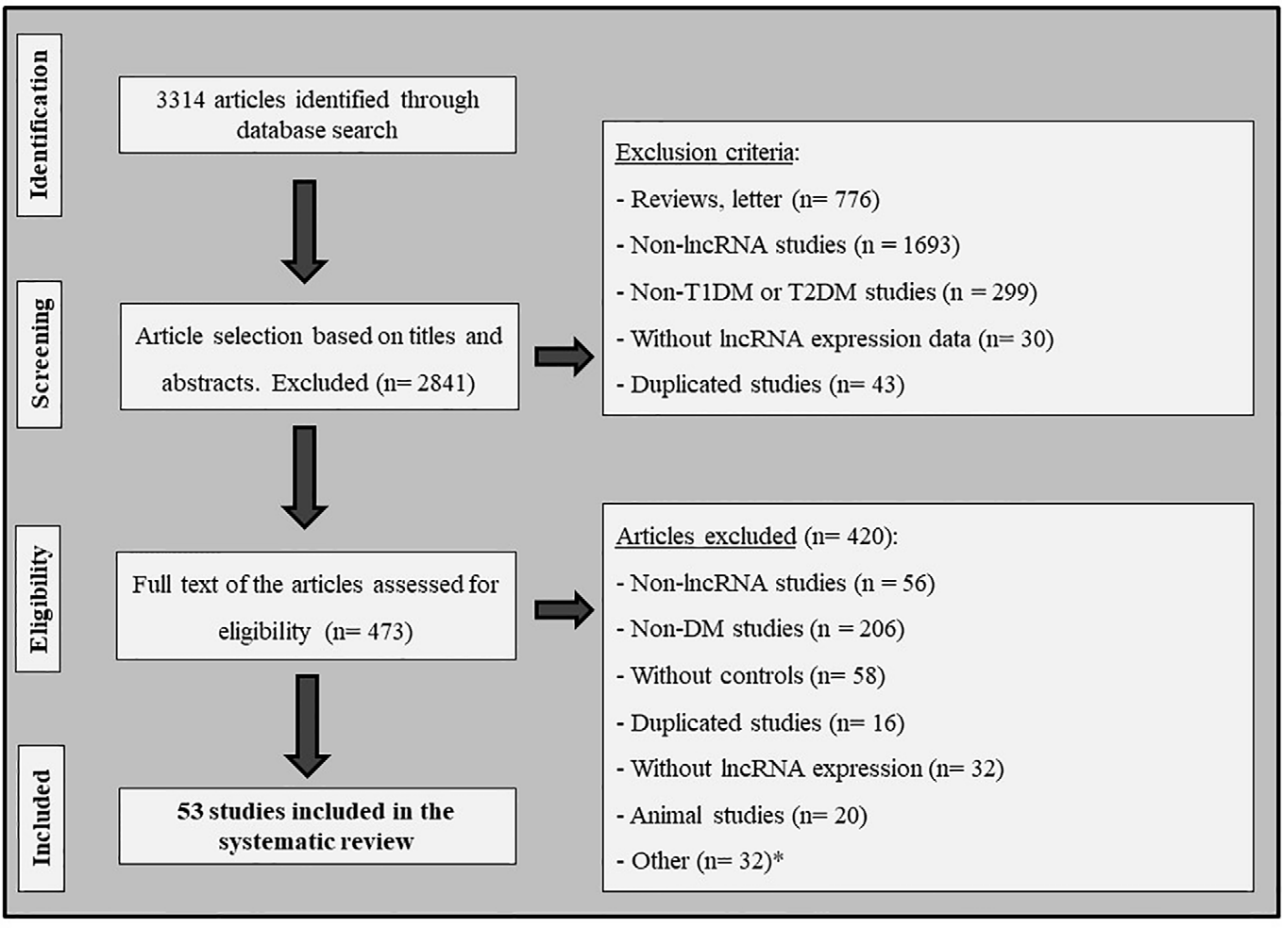
Flowchart illustrating the search strategy used to identify studies that investigated the association between lncRNAs and diabetes mellitus. *Other: articles excluded due to lack of important information; studies with cell lines; and studies written in other idioms (not English, Spanish or Portuguese).

**Figure 1** shows the flowchart illustrating the strategy used to identify and select articles for inclusion in this systematic review. Following the search criteria, a total of 3,314 publications were retrieved from databases; however, after careful full text analysis, only 53 articles fulfilled the eligibility criteria and were included in the present review. The main characteristics of these studies are shown in **Table 1** and the **Supplementary Table 1**.

**Table 1:** Characteristics of studies included in the systematic review.

Author, year [Reference]	Sample size Case/Control	Tissue	Method	Total number of studied lncRNAs	Statistically significant lncRNAs	
					Upregulated	Downregulated
Akerman et al. 2017 (17)	10 T2DM patients/50 controls	Pancreatic islets	RNA-seq and qPCR	2,373	0	16
Alikhah et al. 2018 (36)	18 T2DM patients/18 controls	PBMCs	qPCR	1	0	0
Carter et al. 2015 (23)	5 T2DM patients/5 controls 47 T2DM patients/49 controls (validation)	Serum	Microarray and qPCR	84	0	1
Chen et al. 2019 (37)	25 DM patients/20 controls	Serum	qPCR	1	0	0
Chen et al. 2018 (38)	27 DM patients/17 controls	Serum	qPCR	1	0	0
Cheng et al. 2019 (39)	30 DM patients/30 controls	Peripheral blood	qPCR	1	1	0
Dai et al. 2020 (40)	60 T2DM patients/60 controls	Plasma	qPCR	1	0	0
Das et al. 2018 (41)	5 T2DM patients/5 controls	CD14+ monocytes	qPCR	1	1	0
De Gonzalo-Calvo et al. 2016 (42)	48 T2DM patients/12 controls	Serum	qPCR	12	1	3
Erfanian Omidvar et al. 2019 (24)	100 T2DM patients/100 controls	PBMCs	qPCR	2	0	2
Esguerra et al. 2020 (43)	9 T2DM patients/10 controls	Pancreatic islets	qPCR	1	1	0
Fadista et al. 2014 (44)	12 T2DM patients/51 controls	Pancreatic islets	RNA-seq	493	NA	NA
Fawzy et al. 2020 (45)	53 T2DM patients/110 controls	Plasma	qPCR	2	1	1
Gao et al. 2014 (46)	5 T2DM patients/4 controls	Lateral quadriceps muscle biopsy	qPCR	1	0	1
Jiao et al. 2019 (47)	43 DM patients/48 controls	Serum	qPCR	1	1	0
Kameswaran et al. 2014 (48)	4 T2DM patients/3 controls	Pancreatic islets	qPCR	1	0	1
Li et al. 2018 (49)	10 T2DM patients/10 controls	Liver biopsy	qPCR	1	1	0
Li et al. 2019 (50)	56 T2DM patients/40 controls	Serum	qPCR	1	0	0
Li et al. 2018 (51)	63 DM patients/56 controls	Plasma	qPCR	1	0	0
Li et al. 2018 (25)	6 T2DM patients/6 controls 20 T2DM patients/20 controls (validation)	Peripheral blood	Microarray and qPCR	41,000	14	3
Liu et al. 2019 (52)	90 T2DM patients/30 controls	Serum	qPCR	1	1	0
Luo et al. 2018 (53)	6 T2DM patients/6 controls 26 T2DM patients/26 controls (validation)	PBMCs	Microarray and qPCR	NA	316	126
Ma et al. 2020 (54)	5 T2DM patients/5 controls 122 T2DM patients/125 controls (validation)	PBMCs	Array and qPCR	41,000	44	24
Mansoori et al. 2018 (26)	100 T2DM patients/100 controls	PBMCs	qPCR	2	0	2
Mohamadi et al. 2019 (55)	100 T2DM patients/100 controls	PBMCs	qPCR	2	0	0
Móran et al. 2012 (56)	16 T2DM patients/19 controls	Pancreatic islets	qPCR	13	1	1
Motterle et al. 2017 (57)	10 T2DM patients/10 controls	Pancreatic islets	qPCR	1	0	1
Pengyu et al. 2020 (58)	4 T2DM patients/4 controls	Serum	RNAseq and qPCR	NA	68763	28523
Pradas-Juni et al. 2020 (59)	4 T2DM patients/4 controls	Liver	RNAseq	13,805	126	384
Reddy et al. 2014 (60)	4 T2DM patients/4 controls	Monocytes	qPCR	1	1	0
Ren et al. 2019 (61)	178 T2DM patients/44 controls	Plasma	qPCR	1	0	0
Ruan et al. 2018 (19)	3 T2DM patients/3 controls 30 T2DM patients/30 controls (validation)	Blood	Microarray and qPCR	40,914		2269
	30 T2DM patients/30 controls	Exosome serum/exosome-free serum	qPCR	1	1	0
Saeidi et al. 2018 (27)	100 T2DM patients/100 controls	PBMCs	qPCR	2	0	2
Sathishkumar et al. 2018 (21)	30 T2DM patients/32 controls	PBMCs	qPCR	17	13	2
Shaker et al. 2019 (62)	30 T2DM patients/81 controls	Blood	qPCR	2	2	0
Toraih et al. 2019 (63)	55 T2DM patients/108 controls	Plasma	qPCR	4	4	0
Wan et al. 2020 (64)	32 T2DM patients/32 controls	Serum	qPCR	1	1	0
Wang et al. 2018 (65)	296 T2DM patients/56 controls	Serum	qPCR	1	0	0
Wang et al. 2018 (66)*	2 T2DM patients/2 controls	Blood	Microarray and qPCR	NA	NA	NA
Wang et al. 2017 (28)	6 T2DM patients/6 controls 60 T2DM patients/60 controls (validation)	Peripheral blood	Microarray and qPCR	NA	39	16
Wang et al. 2020 (67)	156 T2DM/100 controls	Peripheral blood	qPCR	3	3	0
Yang et al. 2018 (68)	8 DM patients/8 controls	Serum	qPCR	1	1	0
Yang et al. 2018 (69)	6 DM patients/6 controls	Serum	qPCR	1	1	0
Yang et al. 2018 (70)	36 DM patients/41 controls	Serum	qPCR	1	0	0
Yang et al. 2019 (71)	DM patients/controls	Serum	Array	30,586	245	680
Yin et al. 2019 (72)	62 DM patients/48 controls	Plasma	qPCR	1	0	0
Zha et al. 2019 (73)	244 T2DM patients/126 controls	Plasma	qPCR	1	0	1
Zhang et al. 2018 (74)	28 DM patients/30 controls	Serum	qPCR	1	0	1
Zhang et al. 2020 (75)	99 T2DM patients/50 controls	Serum	qPCR	1	0	1
Zhang et al. 2017 (76)	30 DM patients/28 controls	Plasma	Microarray	NA	NA	NA
Zhang et al. 2019 (77)	24 T2DM patients/26 controls	Serum	qPCR	1	1	0
Zhang et al. 2019 (78)	244 T2DM patients/102 controls	Plasma	qPCR	1	0	0
Zhang et al. 2019 (79)	60 DM patients/60 controls	Plasma	qPCR	1	0	0

*Abstract from congress. DM, diabetes mellitus; NA, information not available; PBMCs, Peripheral blood mononuclear cells; qPCR, quantitative real time PCR; RNA seq, RNA sequencing; T2DM, type 2 diabetes mellitus.

The number of lncRNAs differentially expressed between case and control groups from the different included studies varied from 1 (23, 39, 41, 43, 46–49, 52, 57, 60, 64, 68, 69, 73–75, 77) to 97,286 (58), and the sample sizes ranged from 4 (66) to 370 (73). Among the 53 studies included in this systematic review, 74% of them analyzed T2DM patients, while 26% did not report which DM type patients had. The tissues most analyzed were serum, plasma, and peripheral blood mononuclear cells (PBMCs).

### Differentially Expressed lncRNAs in DM

As shown in the **Supplementary Table 2**, 623 lncRNAs were reported as being dysregulated in patients with DM from one study (17, 21, 24–28, 41, 42, 44, 47, 54, 55, 57–60, 64, 73, 75), while only seven were dysregulated in cases in two studies (*ENST00000550337.1*, *Pluto*, *LncRNAp3134*, *n335556*, *n336109*, *n342533*, and *Pvt1*) (17, 19, 21, 25, 28, 63, 66, 67). Eight lncRNAs were dysregulated in patients from three or more studies, being chosen for further evaluation (**Supplementary Table 2** and **Table 2**). Among these eight lncRNAs, those showing concordant results in more than 75% of the studies were considered consistently dysregulated in DM. Thus, as shown in **Table 2**, six lncRNAs were consistently dysregulated in patients with DM (upregulated: *Anril*, *Hotair*, *Malat1*, *Miat*, and *Kcnq1ot1*; downregulated: *Meg3*) compared to controls. *GAS5* and *H19* were upregulated in patients from some studies and downregulated in others, which could be explained by differences in the tissue types analyzed (serum, pancreatic islets, liver, plasma, and PBMCs) (**Table 2**).

**Table 2:** LncRNAs differentially expressed in at least three studies included in the systematic review.

LncRNA	Reference	Samples	Tissue	Change of expression
*ANRIL*	Sathishkumar et al. (21)	T2DM patients	PBMCs	Up
	Toraih et al. (63)	T2DM patients	Plasma	Up
	Zhang and Wang (77)	T2DM patients	Serum	Up
*GAS5*	Carter et al. (23)	T2DM patients	Serum	Down
	Esguerra et al. (43)	T2DM patients	Pancreatic islets	Up
	Sathishkumar et al. (21)	T2DM patients	PBMCs	Up
*H19*	Cheng et al. (39)	T2DM patients	Peripheral blood	Up
	Fawzy et al. (45)	T2DM patients	Plasma	Up
	Gao et al. (46)	T2DM patients	Muscle	Down
*HOTAIR*	Li et al. (49)	T2DM patients	Liver	Up
	Sathishkumar et al. (21)	T2DM patients	PBMCs	Up
	Shaker et al. (62)	T2DM patients	Blood	Up
*Kcnq1ot1*	Móran et al. (56)	T2DM patients	Pancreatic islets	Up
	Yang et al. (68)	DM patients	Serum	Up
	Yang et al. (69)	DM patients	Serum	Up
*MALAT1*	Liu et al. (52)	T2DM patients	Serum	Up
	Luo et al. (53)	T2DM patients	Blood	Up
	Sathishkumar et al. (21)	T2DM patients	PBMCs	Up
	Shaker et al. (62)	T2DM patients	Blood	Up
	Toraih et al. (63)	T2DM patients	Plasma	Up
*MEG3*	Kameswaran et al. (48)	T2DM patients	Pancreatic islets	Down
	Luo et al. (53)	T2DM patients	Blood	Down
	Sathishkumar et al. (21)	T2DM patients	PBMCs	Up
	Zhang et al. (74)	DM patients	Serum	Down
*MIAT*	De Gonzalo-Calvo et al. (42)	T2DM patients	Serum	Up
	Sathishkumar et al. (21)	T2DM patients	PBMCs	Up
	Toraih et al. (63)	T2DM patients	Plasma	Up

DM, diabetes mellitus; PBMCs, Peripheral blood mononuclear cells; T2DM, type 2 diabetes mellitus.

### Putative Target Genes and Enrichment Pathway Analysis of the Six Differentially Expressed lncRNAs in Human Samples

**Figure 2:**
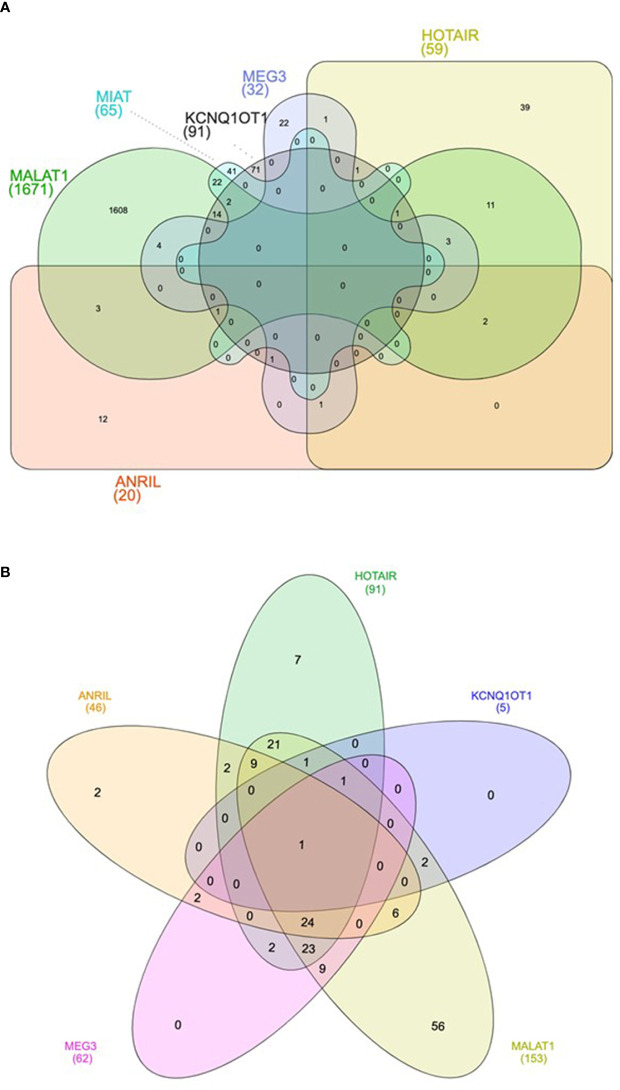
Venn diagram showing the shared target genes **(A)** and pathways **(B)** of the six lncRNAs consistently dysregulated in DM.

Bioinformatics analyses were carried out to find putative targets and biological pathways regulated by the six lncRNAs (*Anril*, *Hotair*, *Malat1*, *Miat*, *Kcnq1ot1*, and *Meg3*) consistently dysregulated in samples of DM patients. These six lncRNAs regulate together the expression of 1,860 unique target genes (**Supplementary Table 3**). *Malat1* has the largest number of target genes (1,671), followed by *Kcnq1ot1* (91), *Miat* (65), and *Hotair* (59), while *Meg3* and *Anril* have the lowest number of targets (32 and 20, respectively) (**Figure 2A** and **Supplementary Table 3**). Among the 1,860 target genes, 1,307 were protein coding genes, 287 were pseudogenes, 100 were small nuclear RNAs (snRNAs), and 225 were other type of ncRNAs, including microRNAs, rRNA, tRNA, and mitochondrial RNA (mtRNA) (**Supplementary Table 3**).

**Figure 3:**
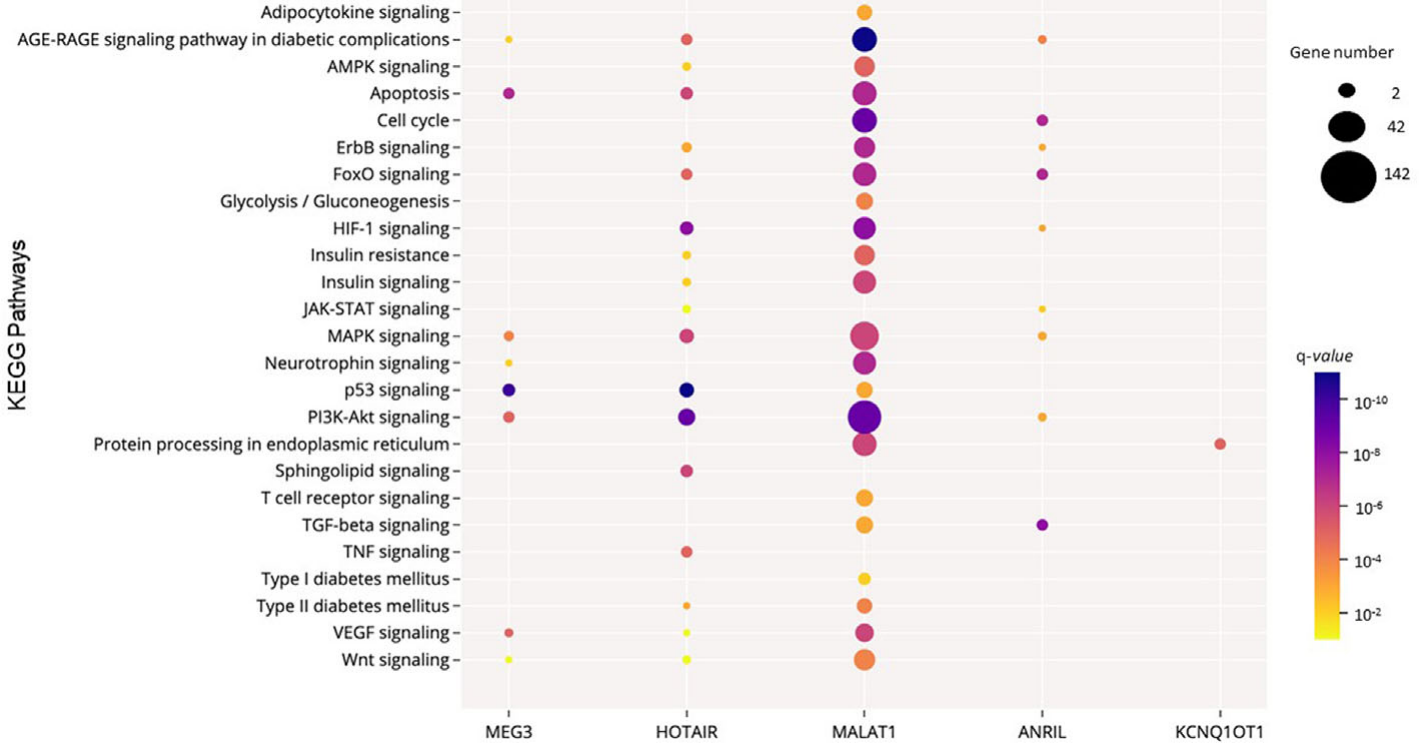
Significant KEGG pathways potentially regulated by the consistently dysregulated lncRNAs in DM. The size and the color of the dots represent the gene number and the range of the pathway’s q-value, respectively. The y-axis represents the KEGG pathways, and the x-axis shows the five lncRNAs that participated in each selected pathway. *MIAT* was not significantly enriched in these selected pathways. Q-*values*: P-*values* corrected for multiple tests using the Benjamini–Hochberg method.

Next, to further explore the functional consequences of the dysregulation of the six lncRNAs of interest, we performed functional enrichment analysis of their protein-encoding target genes using pathways maps from the KEGG repository. As a result, a total of 168 unique pathways were enriched for lncRNA target genes (**Supplementary Table 4**). Moreover, as demonstrated in **Figure 2B**, only one pathway is shared among the five lncRNAs (*Anril*, *Hotair*, *Malat1*, *Kcnq1ot1*, and *Meg3*): Kaposi sarcoma-associated herpes virus infection. Many of the 168 pathways are well established to be involved in DM pathogenesis, such as PI3K/Akt, MAPK, apoptosis, AGE/RAGE, and FoxO (**Figure 3** and **Supplementary Table 4**). Of note, we could not find any significant KEGG pathway for *Miat*.

## Discussion

Currently, several studies have reported the association between epigenetic mechanisms and DM development [reviewed in (6, 7, 80, 81)]. In this context, lncRNAs are a class of ncRNAs that appear to be involved in DM pathogenesis (10). Thus, here, we performed a systematic review to further investigate which lncRNAs are mainly associated with DM. Our results demonstrated six lncRNAs were consistently dysregulated in patients with DM. *Anril*, *Hotair*, *Kncq1ot1*, *Malat1*, and *Miat* were consistently upregulated, while *Meg3* was downregulated in diabetic cases compared to controls.

*Malat1* (metastasis‐associated lung adenocarcinoma transcript 1, also known as *Neat2*) is one of the most analyzed lncRNAs in T2DM samples. Here, our qualitative analysis shows this lncRNA is upregulated in serum, plasma, and PBMCs of T2DM patients (21, 52, 53, 62, 63). Moreover, studies performed in animal models of DM indicate that the expression of *Malat1* is increased in liver, macrophages, and serum of different murine models of T2DM compared to controls (20, 27, 52). *Malat1* is a highly conserved nuclear lncRNA initially identified as a predictor of lung cancer metastasis (82). Several studies have reported the involvement of this lncRNA in signaling pathways related to DM pathogenesis, such as PI3K/Akt (83), NF-κB (84), MAPK/ERK (85, 86), and Wnt/β-catenin (87). Accordingly, our *in silico* analysis shows *Malat1* is involved in a number of pathways involved in DM and its complications that, besides PI3K/Akt, MAPK, and Wnt, include apoptosis, insulin, cell cycle, AMPK, FoxO, ErbB, HIF-1, AGE/RAGE, adipocytokines, and protein processing in endoplasmic reticulum. In agreement with *Malat1* upregulation in T2DM, its expression was also increased in human umbilical vein endothelial cells (HUVECs) cultured with high-glucose (HG) and positively correlated with inflammatory cytokine (IL6 and TNF) levels (88). Additionally, this lncRNA was upregulated in mice with diabetic retinopathy (DR) compared to control animals (89).

*Hotair* was also consistently upregulated in liver, blood, and PBMCs of patients with T2DM (21, 38, 62). Accordingly, Li et al. reported this lncRNA was upregulated in liver of two T2DM murine models (db/db and C57BL/6J mice) treated with high-fat diet (49). *Hotair* is located within the *HOMEOBOX C* (*HOXC*) gene cluster on chromosome 12q13.13 and is involved in cellular proliferation, inhibition of apoptosis, genomic instability, angiogenesis, and metastasis (90–92). Moreover, *Hotair* upregulation promotes hepatic insulin resistance *via* the Akt/GSK pathway (38), which might partially explain its association with T2DM. Our *in silico* analysis demonstrates the involved of *Hotair* in several DM-related pathways, such apoptosis, PI3K-Akt, MAPK, HIF-1, TNF, and FoxO. This lncRNA seems also to be involved in the pathogenesis of diabetic chronic complications. *Hotair* was upregulated in serum of patients with different degrees of DR compared to healthy controls, and its expression was able to distinguish patients with non-proliferative DR from those with proliferative DR (62). Increased expression of *Hotair* was also found in kidney of patients with diabetic kidney disease (DKD) and in kidneys of *db/db* and STZ-induced diabetic mice (93). Accordingly, mouse podocytes cultured under HG conditions also expressed high levels of *Hotair* (93).

In addition to *Malat1* and *Hotair*, the lncRNA *Anril* was also increased in PBMCs, plasma, or serum of patients with T2DM compared to controls (21, 63, 77). This lncRNA has been associated with several types of cancer, such as gliomas, breast, lung, liver, colon, and thyroid cancers [reviewed in (94)]. *Anril* seems also to be involved in DR pathogenesis, since its expression was upregulated in human retinal endothelial cells (HRECs) cultured under HG conditions and in retinal tissue of STZ-induced diabetic mice (95). Blockade of *Anril* prevented HG-induced *VEGF* upregulation in HRECs, which is a key angiogenic factor in DR pathogenesis (95, 96). In line with these findings, Zhang et al. showed *Anril* overexpression in diabetic rats complicated with cerebral infarction upregulated VEGF and improved angiogenesis through activation of the NF-κB pathway (97). Our *in silico* analysis indicates that *Anril* is also involved in the TGFβ, PI3K-Akt, MAPK, cell cycle, FoxO, and AGE/RAGE pathways, which are known pathways related to DM and its chronic complications.

*Kcnq1ot1* is another lncRNA consistently upregulated in islets and serum of patients with T2DM (56, 68, 69). *Kcnq1ot1* is an antisense lncRNA that seems to regulate the expression of both neighboring or distant genes (98), including the *CDKN1C*, a known regulator of beta-cell development (99). Interestingly, a meta-analysis study, including 51,075 DM cases and 10,6134 controls, demonstrated the association between the rs231362 polymorphism in the *Kcnq1ot1* gene and risk for T2DM [OR 1.10 (95% CI 1.06–1.15), P < 10^−4^] (100). Our *in silico* analysis indicates this lncRNA regulates genes from the protein processing in endoplasmic reticulum stress pathway.

*Miat* was also consistently upregulated in serum, plasma, or PBMCs of T2DM patients compared to controls (21, 42, 63). This lncRNA seems to act as a regulator of several signaling pathways related to cellular function, such as proliferation and apoptosis and as a competitive endogenous RNA (101). Additionally, *Miat* seems to be involved in diabetic complications (102). *Miat* was upregulated in the myocardium of diabetic rats, while its knockdown inhibited apoptosis in cardiomyocytes exposed to HG (103). In contrast, in renal tubuli of diabetic rats, *Miat* was downregulated compared to control rats and negatively correlated to serum creatinine levels (104). Growing evidence has also shown *Miat* dysregulation in a number of diseases, such as myocardial infarction, age-related cataract, different cancers, and ischemic stroke [reviewed in (101)]. Here, we were not able to find any significant KEGG pathway for *Miat*; therefore, how this lncRNA is involved in DM and other diseases still needs to be clarified.

Our systematic review indicates *Meg3* is downregulated in islets, whole blood, and serum of patients with DM (48, 53, 74). Accordingly, this lncRNA was downregulated in islets of db/db mice (105) and in serum of diabetic patients with DR compared to controls (74). However, it was upregulated in liver or primary hepatocytes of different T2DM murine models (59, 106). In a murine beta-cell line (MIN6), *Meg3* suppression led to increased apoptosis due to *caspase-3* and *Bax* upregulation and *Bcl2* downregulation (105). In addition, *Meg3* seems to regulate insulin synthesis and secretion since its blockade in murine beta-cells decreased the expression of key transcription factors involved in insulin synthesis (Pdx-1 and mafA); thus, decreasing insulin gene transcription (105). Besides apoptosis, our *in silico* analysis suggests this lncRNA is involved in PI3K/Akt, VEGF, and MAPK pathways.

Of note, our bioinformatics analysis also demonstrated that *Anril*, *Hotair*, *Malat1*, *Kcnq1ot1*, and *Meg3* regulate genes from the Kaposi sarcoma-associated herpes virus infection (KSHV) pathway. KSHV, also known as human herpesvirus 8, is a human tumor virus associated with the pathogenesis of Kaposi’s sarcoma, primary effusion lymphoma, and Multicentric Castleman’s disease. The KSHV pathway contains genes related to IFN antiviral response, inflammatory cytokines, and cell proliferation pathways [https://www.genome.jp/kegg/kegg2.html]. Interestingly, the association between KSHV and DM was previously reported by observational studies (107, 108). Cui et al. described that patients with T2DM had an elevated risk of KSHV (107). Accordingly, Piras et al. showed 58% of T2DM patients were seropositive for KSHV *vs.* 27% of the healthy subjects (108). Even though the mechanisms behind this association are unknown, this virus causes metabolic changes that might lead to altered insulin uptake and accumulation of neutral lipids in cells and also induce an impairment of the immune system [review in (109)], which are mechanisms related to DM pathogenesis.

Even though this systematic review indicates a group of lncRNAs consistently associated with DM and the pathways possible regulated by them, it has few limitations. First, there is no official nomenclature for lncRNAs; thus, we cannot exclude the possibility that we have lost some information. Second, some studies, especially those using RNAseq and microarrays technologies, did not inform which were the differentially expressed lncRNAs or their expression pattern (up- or downregulation) (19, 25, 44, 53, 54, 58, 66, 71, 76). Third, studies used different techniques to quantify lncRNA expressions and usually did not provide the expression values, only the pattern of expression of the dysregulated lncRNAs; therefore, making impossible to perform a reliable quantitative analysis of the data (meta-analysis). Fourth, most of the studies investigated lncRNAs in patients with T2DM or did not inform the type of DM, evidencing the lack of studies in T1DM population. In this context, four of the dysregulated lncRNAs found in this study were analyzed only in T2DM patients (*Anril*, *Hotair*, *Malat1*, and *Miat*). Thus, our results are more representative of this type of DM. Fifth, although six lncRNAs were consistently dysregulated in patients with DM compared to controls, it was not possible to perform a stratified analysis by tissue type since the number of studies that evaluated the same lncRNA in a given tissue is very small. Lastly, as commented above, *Anril*, *Hotair*, *Kcnq1ot1*, *Malat*, *Meg3*, and *Miat* lncRNAs seem to be dysregulated in patients with DR and DKD. However, most of the studies included in this systematic review did not report the percentage of patients with these diabetic chronic complications. Thus, here, it was impossible to evaluate if presence of diabetic chronic complications is impacting our results. Further studies are required to clarify this point.

In conclusion, our systematic review indicates that six lncRNAs are consistently dysregulated in DM, especially in patients with T2DM. This study also contributes to enlighten the pathways regulated by these lncRNAs and involved in the DM pathogenesis, such as PI3K/Akt, MAPK, apoptosis, AGE/RAGE, and FoxO. Although this systematic review included 53 studies which analyzed lncRNA expression in DM-related tissues, further studies are necessary to better understand the involvement of lncRNAs in the pathogenesis of this complex disease and its chronic complications. As much as lncRNAs seem to be good candidates as biomarkers and therapeutic targets for DM, further investigations on organ-specific distribution of these regulatory molecules may be useful to clarify their role in DM.

## Data Availability Statement

The raw data supporting the conclusions of this article will be made available by the authors, without undue reservation.

## Author Contributions

CD designed the study, researched data, performed the analysis, and wrote the manuscript. NL researched data, performed the analysis, and reviewed the manuscript. NC researched data and reviewed the manuscript. TA researched data, performed the bioinformatics analyses, contributed to discussion, and reviewed the manuscript. DC designed the study, contributed to the discussion, and wrote and reviewed the manuscript. All authors contributed to the article and approved the submitted version.

## Funding

This study was partially supported by grants from the Conselho Nacional de Desenvolvimento Científico e Tecnológico (CNPq), Fundo de Incentivo à Pesquisa e Eventos (FIPE, number 2018-0470) at Hospital de Clínicas de Porto Alegre, Fundação de Amparo à Pesquisa do Estado do Rio Grande do Sul (FAPERGS) (Edital FAPERGS/CNPq 12/2014 PRONEX - Processo n° 16/2551 - 0000483-8), and Coordenação de Aperfeiçoamento de Pessoal de Nível Superior (CAPES). DC is recipient of scholarships from CNPq, while CD and TSA are recipients from scholarships from CAPES, and NL is recipient of scholarships from FAPERGS.

## Conflict of Interest

The authors declare that the research was conducted in the absence of any commercial or financial relationships that could be construed as a potential conflict of interest.
