# Supplementary material for: The Impact of lncRNAs in Diabetes Mellitus: A Systematic Review and In Silico Analyses
Source: Front Endocrinol (Lausanne). 2021 Mar 19;12:602597. doi: 10.3389/fendo.2021.602597 (PMC8018579; doi:10.3389/fendo.2021.602597)
Supplement: Supplementary file 1 [file Table_1.docx]

**Supplementary Table 1.** Characteristics of patients of the studies included in the systematic review.

| **Author, year [Reference]** | **Sample size**  **Case/Control** | **Tissue** | **Gender (% males)**  **Case/control** | | **Age (case/control)** | | | **Country** |
| --- | --- | --- | --- | --- | --- | --- | --- | --- |
| Akerman, 2017 [17] | 10 T2DM patients */* 50 controls | Pancreatic islets | 55.6 / 53.7 | 57.0 ± 4.0 / 59.0 ± 9.0 | | | Sweden | |
| Alikhah, 2018 [95] | 18 T2DM patients / 18 controls | PBMCs | NA | NA | | | Iran | |
| Carter, 2015 [23] | 5 T2DM patients / 5 controls  47 T2DM patients / 49 controls (validation) | Serum | NA | 70.3 ± 9.1 / 66.9 ± 9.7 | | | United States | |
| Chen, 2019 [96] | 25 DM patients/ 20 controls | Serum | 48.0 / 50.0 | 59.5 ± 9.0 / 55.7 ± 10.1 | | | China | |
| Chen, 2018 [74] | 27 DM patients/ 17 controls | Serum | 51.8 / 52.9 | 44.4 ± 5.7 / 41.9 ± 6.2 | | | China | |
| Cheng, 2019 [36] | 30 DM patients/ 30 controls | Peripheral blood | NA | NA | | | China | |
| Dai, 2020 [97] | 60 T2DM patients/ 60 controls | Plasma | 63.3 / 63.3 | 44.2 ± 5.0 / 44.3 ± 5.1 | | | China | |
| Das, 2018 [37] | 5 T2DM patients / 5 controls | PBMCs | NA | NA | | | United States | |
| De Gonzalo-Calvo, 2016 [59] | 48 T2DM patients / 12 controls | Serum | NA | 57.5 ± 5.4 / 57.7 ± 6.7 | | | Netherlands | |
| Erfanian Omidvar, 2019 [24] | 100 T2DM patients/ 100 | PBMCs | 52.0 / 65.0 | 54.5 ± 8.7 / 52.2 ± 8.5 | | | Iran | |
| Esguerra, 2020 [38] | 9 T2DM patients / 10 controls | Pancreatic islets | 40.0 /50.0 | 49.3 / 56.9 | | | Sweden | |
| Fadista, 2014 [56] | 12 T2DM patients / 51 controls | Pancreatic islets | 50.0 / 64.7 | 61.0 ± 10.0 / 56.0 ± 12.0 | | | Sweden | |
| Fawzy, 2020 [98] | 53 T2DM patients / 110 controls | Plasma | 79.2 / 25.5 | 62.6 ± 7.3 / 60.5 ± 10.7 | | | Egypt | |
| Gao, 2014 [39] | 5 T2DM patients / 4 controls | Lateral quadríceps muscle biopsy | 40.0 / 25.0 | 63.0 / 24.7 | | | China | |
| Jiao, 2019 [40] | 43 DM patients / 48 controls | Serum | 55.8 / 56.2 | 48.4 ± 7.1 / 48.1 ± 6.2 | | | China | |
| Kameswaran, 2014 [41] | 4 T2DM patients / 3 controls | Pancreatic islets | 50.0 / 33.3 | 53.2 / 35.3 | | | United States | |
| Li, 2018 [51] | 10 T2DM patients / 10 controls | Liver biopsy | 50.0 / 60.0 | 45.6 ± 7.4 / 48.2 ± 4.2 | | | China | |
| Li, 2019 [99] | 56 T2DM patients/ 40 controls | Serum | 53.6 / 65.0 | 44.8 ± 5.2 / 46.0 ± 6.4 | | | China | |
| Li, 2018 [100] | 63 DM patients / 56 controls | Plasma | 57.1 / 57.1 | 46.1 ± 6.5 / 45.3 ± 7.2 | | | China | |
| Li, 2017 [25] | 6 T2DM patients / 6 controls  20 T2DM patients / 20 controls (validation) | Peripheral blood | NA | NA | | | China | |
| Liu, 2019 [47] | 90 T2DM patients / 30 controls | Serum | 62.2 / 53.3 | 55.5 ± 9.8 / 53.6 ± 9.2 | | | China | |
| Luo, 2018 [64] | 6 T2DM patients / 6 controls  26 T2DM patients / 26 controls (validation) | PBMCs | NA | NA | | | China | |
| Ma, 2020 [57] | 5 T2DM patients / 5 controls  122 T2DM patients / 125 controls (validation) | PBMCs | NA | 54.3 ± 9.8 / 48.3 ± 10.2 | | | China | |
| Mansoori, 2018 [26] | 100 T2DM patients / 100 controls | PBMCs | NA | 60.9 ± 0.9 / 58.1 ± 1.2 | | | Iran | |
| Mohamadi, 2019 [58] | 100 T2DM patients / 100 controls | PBMCs | 52.0 / 65.0 | 54.5 ± 8.7 / 52.2 ± 8.5 | | | Iran | |
| Móran, 2012 [83] | 16 T2DM patients / 19 controls | Pancreatic islets | 37.5 / 50.0 | 55.5 / 49.2 | | | France | |
| Motterle, 2017 [42] | 10 T2DM patients / 10 controls | Pancreatic islets | 50.0 / 50.0 | 55.5 ± 3.0 / 56.9 ± 2.5 | | | Switzerland | |
| Pengyu, 2020 [53] | 4 T2DM patients / 4 controls | Serum | NA | NA | | | China | |
| Pradas-Juni, 2020 [55] | 4 T2DM patients / 4 controls | Liver | 100.0 / 100.0 | 75.5 ± 0.7 / 60.7 ± 9.0 | | | Germany | |
| Reddy, 2014 [43] | 4 T2DM patients / 4 controls | Monocytes | NA | 43.2 ± 6.3 / 37.5 ± 3.8 | | | United States | |
| Ren, 2019 [101] | 178 T2DM patients / 44 controls | Plasma | 53.7 / 61.3 | 46.2 ± 6.2 / 46.7 ± 5.5 | | | China | |
| Ruan, 2018 [19] | 3 T2DM patients / 3 controls  30 T2DM patients / 30 controls (validation) | Blood | 66.6 / 46.6 | 42.2 ± 9.7 / 48.9 ± 10.5 | | China | | |
|  | 30 T2DM patients / 30 controls | Exosome serum/ exosome-free serum |  |  | | |  | |
| Saeidi, 2018 [27] | 100 T2DM patients/ 100 controls | PBMCs | 36.0 / 35.0 | 60.90± 0.9 / 58.1 ± 1.2 | | | Iran | |
| Sathishkumar, 2018 [21] | 30 T2DM patients /32 controls | PBMCs | 56.2 / 60.0 | 46 ± 8 / 44 ± 8 | | | India | |
| Shaker, 2019 [65] | 30 T2DM patients / 81 controls | Blood | 63.3 / 66.6 | 51.0 ± 6.1 / 50.7 ± 7.7 | | | Egypt | |
| Toraih, 2019 [60] | 55 T2DM patients/ 108 controls | Plasma | 26.9 / 56.5 | 60.8 ± 8.7 / 59.2 ± 5.6 | | | Egypt | |
| Wan, 2020 [44] | 32 T2DM patients / 32 controls | Serum | 59.4 / 62.5 | 45.1 ± 5.6 / 43.5 ± 6.6 | | | China | |
| Wang, 2018 [102] | 296 T2DM patients / 56 controls | Serum | 56.1 / 53.6 | 47.4 ± 6.1 / 49.5 ± 7.7 | | | China | |
| Wang, 2018 [54]* | 2 T2DM patients / 2 controls | Blood | NA | NA | | | China | |
| Wang, 2017 [28] | 6 T2DM patients / 6 controls  60 T2DM patients / 60 controls (validation) | Peripheral blood | 61.7 / 58.3 | 50.4 ± 13.4 / 51.0 ± 9.0 | | | China | |
| Wang, 2020 [61] | 156 T2DM / 100 controls | Peripheral blood | 62.2 / 57.0 | 53.3 ± 11.7 / 51.8 ± 9.6 | | | China | |
| Yang, 2018 [52] | 8 DM patients / 8 controls | Serum | NA | NA | | | China | |
| Yang, 2018 [46] | 6 DM patients / 6 controls | Serum | NA | NA | | | China | |
| Yang, 2018 [103] | 36 DM patients / 41 controls | Serum | NA | NA | | | China | |
| Yang, 2019 [94] | DM patients / controls | Serum | NA | NA | | | China | |
| Yin, 2019 [104] | 62 DM patients / 48 controls | Plasma | 54.8 / 47.9 | 47.9 ± 6.9 / 48.8 ± 6.1 | | | China | |
| Zha, 2019 [45] | 244 T2DM patients / 126 controls | Plasma | 56.1 / 53.9 | 48.2±5.6 / 48.9±5.3 | | | China | |
| Zhang, 2018 [48] | 28 DM patients / 30 controls | Serum | 42.8 / 36.6 | 53 ± 14.1 / 54 ± 13.7 | | | China | |
| Zhang, 2020 [49] | 99 T2DM patients / 50 controls | Serum | 50.5 / 54.0 | 53.1 ± 9.2 /50.9 ± 6.8 | | | China | |
| Zhang, 2017 [93] | 30 DM patients / 28 controls | Plasma | 60.0 / 60.7 | 53.2 ± 7.8 / 33.1 ± 10.8 | | | China | |
| Zhang, 2019 [50] | 24 T2DM patients / 26 controls | Serum | NA | NA | | | China | |
| Zhang, 2019 [105] | 244 T2DM patients / 102 controls | Plasma | 54.5 / 53.6 | 46.4 ± 5.5 / 48.1 ± 6.2 | | | China | |
| Zhang, 2019 [106] | 60 DM patients / 60 controls | Plasma | 56.6 / 58.3 | 49.1 ± 6.3 / 51.9 ± 6.7 | | | China | |

*Abstract from congress. DM: diabetes mellitus; NA: not available; PBMCs: Peripheral blood mononuclear cells; T2DM: type 2 diabetes mellitus.
